# Using Facebook for Large-Scale Online Randomized Clinical Trial Recruitment: Effective Advertising Strategies

**DOI:** 10.2196/jmir.9372

**Published:** 2018-11-08

**Authors:** Laura Akers, Judith S Gordon

**Affiliations:** 1 Oregon Research Institute Eugene, OR United States; 2 College of Nursing University of Arizona Tucson, AZ United States

**Keywords:** research subject recruitment, advertisements, social media

## Abstract

Targeted Facebook advertising can be an effective strategy to recruit participants for a large-scale online study. Facebook advertising is useful for reaching people in a wide geographic area, matching a specific demographic profile. It can also target people who would be unlikely to search for the information and would thus not be accessible via Google AdWords. It is especially useful when it is desirable not to raise awareness of the study in a demographic group that would be ineligible for the study. This paper describes the use of Facebook advertising to recruit and enroll 1145 women over a 15-month period for a randomized clinical trial to teach support skills to female partners of male smokeless tobacco users. This tutorial shares our study team’s experiences, lessons learned, and recommendations to help researchers design Facebook advertising campaigns. Topics covered include designing the study infrastructure to optimize recruitment and enrollment tracking, creating a Facebook presence via a *fan page*, designing ads that attract potential participants while meeting Facebook’s strict requirements, and planning and managing an advertising campaign that accommodates the rapid rate of diminishing returns for each ad.

## Introduction

In the past two decades, health interventions via electronic media (electronic health, eHealth) have become widespread. Some studies continue to recruit through traditional methods such as print and broadcast media, flyers, and word-of-mouth, whereas others have taken advantage of online methods such as social media publicity and search engine advertisements.

Facebook has become a major player in the field of digital advertising, with about US $8 billion revenue in 2015 [1]. Facebook allows advertisers to create target audiences by specifying gender, marital status, age, and geographic region as well as other personal characteristics. About 74% of adult American women and 62% of adult American men use Facebook [2]. Facebook is widely used in all age groups (78% of age 30-49 years, 65% of age 50-64 years, and 41% of age 65 years and above) and across racial and ethnic categories (67% of whites, 70% of blacks, and 73% of Hispanics) [2].

Facebook is now widely used in research. In their 2015 *American Psychologist* article, Kosinski et al [3] summarized a wide variety of ways by which social scientists are now using Facebook as a research tool, from recruiting participants (with paid advertising or snowball sampling) to tracking participants across studies and staying connected with participants over time, to collecting and using Facebook profile data, and collecting self-reports. Their detailed discussion of ethical considerations focuses on privacy, consent, and appropriate boundaries. Facebook has also been used to deliver interventions, for example, increasing physical activity among young adult cancer survivors [4] and reducing problem drinking at a university [5]. Whitaker et al [6] reviewed 35 unique studies that used Facebook to recruit participants for health, medical, or psychosocial research and found that the participants were broadly similar to those recruited via traditional methods, although various studies reported an over-representation of women, white people, younger people, and people who were better educated or had a higher rate of income than the general public. Topolovec-Vranic and Natarajan [7] reviewed 30 studies that used both social media and at least one other method for recruitment; of the 14 studies reporting demographic information, 12 studies found that their social media sample was different from those recruited via traditional methods. Frandsen et al [8] showed that participants recruited to their smoking cessation study via social as compared with traditional media were somewhat younger (mean age: 39.3 years vs 44.9 years) but otherwise demographically similar (gender, education, and income). Those recruited via traditional media were more likely to follow the study protocol and complete the study.

To date, most of the studies reporting the use of Facebook recruitment have been surveys [9-17] or modest-sized randomized clinical trials (RCTs) [18-20] and pilot studies [21]. Facebook can also be used to recruit participants for larger-scale RCTs [22,23], but this requires careful planning and oversight to ensure the optimal use of funds allocated for recruitment. Our team relied on Facebook advertising for a 2010 pilot study, recruiting 522 women who were wives or domestic partners of smokeless tobacco users, and we used a similar strategy for a larger RCT, recruiting 1145 participants from the United States and Canada between August 2015 and November 2016. Interested women visited the study website and completed information for eligibility screening, gave informed consent, provided contact information, and completed a baseline survey entirely online. Participating women were then automatically randomized by the Web-based program to receive an intervention (access to an interactive website plus mailed booklet) or delayed treatment (receiving the intervention after completing a 7.5-month follow-up assessment). The intervention program was designed to help the women encourage their partner to consider quitting smokeless tobacco, support him if he decided to do so, and accept his decision if he was not interested in quitting [24]. All study activities were conducted online, with the exception of phone calls to complete follow-up assessments for women who did not respond to email reminders. Ethics approval for the RCT was obtained from the institutional review board at Oregon Research Institute. This study is registered at ClinicalTrials.gov (ID: NCT01885221).

The goal of this paper is to share strategies and *lessons learned* from implementing Facebook advertising to recruit large samples from a narrowly targeted population. As Facebook’s advertising procedures and features change frequently, a specific *how to* article is not feasible. Instead, this paper provides general guidelines and principles that should remain valid for the foreseeable future.

## Steps and Strategies

### Deciding Whether Facebook Advertising Is Right for Your Study

A wide variety of methods exist for recruiting participants for online RCTs, ranging from traditional print and broadcast media and in-person recruitment to innovative uses of social networks (eg, virtual snowball sampling [25]). A comparison of studies recruiting via social media and at least one other method found that for those studies reporting cost-effectiveness, about half found social media to be more cost-effective and about half did not [7]. Many companies offer online paid advertising, and many times, other services will be more cost-effective than Facebook. When recruiting participants for a study topic related to words that potential participants are likely to type into search engines, Google AdWords is an obvious option (eg, for depression prevention [26]). For studies related to specific health conditions, existing online communities may provide research studies with access to their members (although in our experience, many communities now tend to view research as exploitive and will not cooperate). Facebook advertising can be especially useful for studies targeting specific geographical or demographic groups, for conditions for which participants are not already seeking help (eg, using search engines), and for topics for which friends and family would be likely to refer them to the study (ie, by *tagging* them when they see the ad, that is, naming their friend in a comment on the ad in such a manner that the friend receives a notification). Facebook advertising is especially suitable for cases when the study team wishes to avoid exposing a particular demographic group to the advertisements—in our case, we had previously found that men interested in quitting smokeless tobacco would sometimes claim to be women (when undergoing online screening) to get access to our studies for women. To minimize this form of participant fraud, we sought to advertise in locations where only women would see the ad.

### Designing Infrastructure for Enrolling and Managing Participants and for Monitoring Facebook Ads Over Time

At the intervention design stage, we recommend taking several factors into account to facilitate recruitment and enrollment in eHealth studies, which are challenged by the lack of personal contact with participants. It is imperative to have an infrastructure in place that allows easy access for potential participants and real-time monitoring of recruitment strategies linked to participant enrollment. Planning and creating this infrastructure will typically take place many months before study recruitment begins.

First, in our experience, we have found that enrollment for an online study is generally more successful if potential participants are allowed to stay within a single medium, that is, entirely online, rather than asking them to switch from being online to using a phone or mail. Changing from one medium to another may feel more burdensome as it may involve having to create a reminder to do so at a later time (eg, when the person is done with their online activities). In an earlier study, when we switched from asking participants to call a phone number they saw online to registering online directly, enrollment dramatically improved. If phone contact is an important step in enrollment for the study, we recommend that the research team initiate such contact without relying on the potential participant (eg, ask the participant to provide their phone number for the researchers to call).

**Figure 1:**
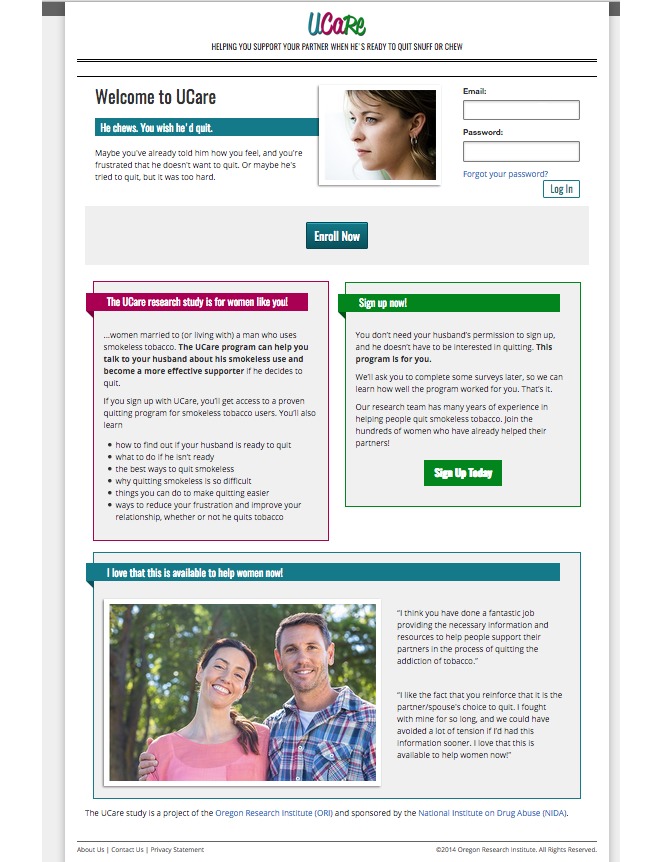
UCare study home page.

Second, the study home page, where the potential participant will go after clicking on the ad, should be pleasing to the eye, maximally welcoming, and aimed at a lay audience. Our first attempt at a study home page was a research-oriented, text-heavy description of the study conditions and procedures. On the basis of poor response from our first few days of recruitment, we overhauled the page, rewriting the text in a friendlier style that focused on the woman’s concerns about her partner and how study participation could benefit her. We added more graphics and user testimonials (see Figure 1). Our conversion of Facebook *clicks* to enrolled participants improved immediately (eg, from 0.5 to 3 participants per day).

Third, as part of the enrollment infrastructure, a study needs either an administrative portal (*admin page*) or other robust participant management system to make it easy to monitor enrollment and the success of recruitment strategies. For maximum usefulness, we recommend that the admin page *dashboard* displays the information needed to monitor the performance of each successive version of the ad (eg, total numbers of people completing each step of the enrollment process) [27], along with a downloadable Excel file of participant-level data for more in-depth tracking and analysis. The participant management system can send an automated email to the research staff at the same time each day with key enrollment figures such as total enrolled overall, total enrolled for each condition, and new enrolled since the previous day. In our study, we used a system custom-developed for us by our software subcontractor, but it may be possible to customize a more generic system for this purpose, such as REDCap.

### Staffing the Advertising Campaign

Assign primary responsibility for the Facebook ad campaign to research staff who are detail-oriented, with good writing and graphic design skills and excellent spelling and punctuation. The research staff should have access to the study’s online administrative functions and authority to use an organizational credit card. These staff will use their personal Facebook accounts to access the Facebook advertising management system (currently called “Ad Manager”). They will need to search their Facebook home page for a link to *Create Ad* or other reference to Facebook advertising; once they have begun working with Facebook ads, Facebook will likely display the link to access the Ad Manager more prominently.

Running the ad campaign will take approximately 1 hour per week, after an initial investment of 1 to 2 hours to learn how the system works and create the initial ad. Subsequent ads can generally be adapted from the first ad, which would typically take 5 to 10 min 2 or 3 times a week. Overall, the time commitment is small, but daily monitoring of the campaign will allow the staff to respond rapidly to changes in the response rate.

### Establishing the Study’s Facebook Presence

Before beginning the advertising campaign, it is important to create a Facebook *fan page* for the study, which will be connected to the ad. Ask friends and colleagues to *like* the page so that it will not look new to potential participants. We gave our page the name “UCare Research Study,” in the category “Community,” with the study home page as the “Website,” and a “Short Description” summarizing how the study could potentially help the participants. Assign at least two staff members (at least one of whom should also have responsibility for the ad campaign) as managers of the page; they will receive Facebook notifications of people’s interactions with both the page and the study’s linked ads.

Keep the fan page active by regularly posting news and related news links to the page, also referred to as *curating* the page. We found that updating the page twice a week was a good rate, balancing the time needed to find and post news items with the desirability of keeping the page content looking fresh. Few of our links received evidence of attention (likes or shares), but their presence makes the page (and by extension, the research study) appear active. If the intervention arm involves providing information to participants or changing their behavior, take care not to reveal or overly hint at the intervention content on this page, as this could potentially contaminate the participants randomized to the control condition.

### Designing the Advertising Campaign

Facebook currently uses an advertising structure of *ads* within *ad sets* within *campaigns*. The *campaign* could encompass all ads for a single study, or multiple campaigns could be created for the study. Combining all ads into 1 campaign makes it easier to monitor the costs. The *ad set* (formerly *advert set*) is where the researcher delineates the criteria of the target audience and the budget; the *ad* (formerly *advert*) is where the graphics and text for the ad are designed. Each of the 3 levels must be switched on for an ad to display. While designing your first ads, keep the overall campaign turned off until you are ready to start advertising.

Plan to create multiple *ad sets*, each targeting a meaningful subsection of your overall audience, such as by geographic region, age group, or gender. Plan to switch your ad from one ad set to another (*rotating* it) when each ad reaches *saturation* (the limit of cost-effective enrollment of participants) and new enrollments trickle to a minimum.

Design the ad campaign with a specific rotation strategy in mind (eg, geographic region or a demographic variable). We began by advertising to our target demographic throughout the United States and Canada, but after 9 days and 12,405 clicks, we had randomized only 3 participants. We then chose to rotate our ad to different geographic regions, targeting 1 region at a time and focusing on areas with high smokeless tobacco use prevalence. All our ads targeted women, aged 25 to 65 years and above, who specified their language as English and whose relationship status was married, engaged, domestic partnership, or in a relationship.

The more specifically you can target your ads, the more cost-effective they will be—if you are paying per click, you will not want to encourage clicks by people ineligible for the study. To this end, Facebook also offers more sophisticated targeting options based on *interests* (eg, *healthy eating* and *bodybuilding*); Facebook gleans this information from information that users provide about themselves, such as Facebook pages they like or Facebook groups they join [28]. In other publications about Facebook advertising, *interests* have also been referred to as *keywords* [10,12,29] and *key profile words* [14].

However, as Pedersen and Kurz [30] note, recruiting based on Facebook interests or *likes* will fail to capture many populations. They give the example of a spouse of a problem drinker who will not *like* Al-Anon groups on Facebook if they do not want their Facebook friends to know their partner is a problem drinker. In our case, there were no specific *interests* that were suitable for our population of women who wanted their partner to quit his use of smokeless tobacco.

Furthermore, one could ask permission to post about a study in a Facebook *group*, a community of people sharing an interest, but approval must be obtained from the group moderator. For our study, there were no organized groups of women concerned about their husbands’ smokeless tobacco use. Studies recruiting people with a specific medical diagnosis or shared health promotion activity may have better luck in finding Facebook groups to target. Groups may be found by typing a keyword into the main Facebook search box and then selecting *groups* to display communities fitting that criterion. Valdez et al [31] described their experiences using Facebook *groups* for recruitment; in 2 studies, the groups yielded 166 participants, which they found sufficient for qualitative but not quantitative research. Our efforts to use Facebook groups to publicize an unrelated study were unsuccessful as many groups now have firm policies against mentioning research opportunities.

Facebook offers the opportunity to pay per impression (every time they show someone the ad) or per click (every time someone clicks on the ad). The former is most suited to cases where the advertiser wants the public to be aware of some information but without taking an immediate action; a good example is a political campaign. The latter is best when you want people to take an action such as clicking on the ad to visit your study home page and consider enrolling. We chose to pay per click. The payment amount for Facebook outcomes such as clicks is determined by a highly complex auction process. We chose the default, *automatic bidding*, which (according to the Ad Manager) will “let Facebook set the bid that helps you get the most link clicks at the best price.”

Decide how much to budget per day for the ad. We budgeted for US $250 per day, based on our previous experience in Facebook advertising with this population, which had cost US $70 per participant. We anticipated that the US $250 per day budget would yield 3 to 4 participants per day and allow us to complete our recruitment in 10 to 11 months. In the summer of 2016, Facebook decided not to observe the daily budgets strictly but to let them average out over time. Assign a credit card to pay for the ads automatically, and be sure that the credit limit is at least twice the amount budgeted per month so the ads can continue while the previous month’s bill is being processed and paid. If it takes longer to pay the bill at your institution, then adjust your credit limit accordingly. After the daily budget is entered into the Ad Manager, it will display the “estimated daily results reach”—the number of people likely to see the ad on a given day. Our numbers were typically 15,000 to 40,000 people, which yielded about 5 participants per day the first time we ran the ad in each location and later yielded 1 to 3 participants per day. When determining their daily advertising budget, researchers should take into account both their study’s desired recruitment period and the risk of sinking costs into an ineffective campaign. It is important not to spend more per day than necessary and yet to spend enough so that effective ads can be distinguished from ineffective ads. The Advertising Costs section below discusses the cost issue more generally.

### Designing the Ad

Facebook ads have strict design specifications that generally involve a graphic image and several categories of text, which are displayed in varying ways depending on where the ad will appear, for example, in the right margin, in the newsfeed, and on Instagram (Instagram, owned by Facebook, may be better for reaching adolescents than Facebook itself [32].). Ad specifications change frequently. When we began advertising for our pilot study in 2010, Facebook ads could be text-only, with an optional image. Later, the image became a requirement and was generally small and square. When we began recruiting for our new study in August 2015, we found that the image was again required and the dimensions must be 1200x628 pixels (a horizontal orientation). In early 2013, Facebook began regulating the amount of text that could appear within the image, and for some time, it had a limit of 20% [33]. As of late 2016, there was no official percentage limit on text within graphics, but any such text (including product logos) was highly discouraged.

For our study, the graphic theme was happy couples, and because such images convey no information about quitting smokeless tobacco, we did include text in our image to help catch the eye of potential participants (our UCare logo and the phrase “a program for women who want a chewer to quit”). We used stock images for our graphics, showing a smiling younger-middle-aged couple, which seemed sufficiently generic to appeal to both younger and older couples, all of whom are in our target audience. The mean age of women in our final sample was 43.2 years (SD 9.5, range 19-78). We also went with white couples for the ad because the vast majority of smokeless users are white.

For the text part of the ad, Facebook currently allows a brief title and a brief block of descriptive text. The text should be succinct and read like an advertisement, without implying that you are trying to sell something. For maximum credibility, have the text mention that recruitment is for a research study conducted by a hospital, university, or nonprofit organization [34]. An ideal ad would encourage those eligible for the study to click the ad and those who are not eligible (or do not have eligible friends) to ignore it. Note, however, that Facebook prohibits advertisement that “asserts or implies personal attributes including disability or medical condition (including physical or personal health).” They prefer that ads say that help for a condition is available than attempt to engage the reader more directly, for example, “Depression counseling available” rather than “Depression getting you down? Get help now.” [35].

**Figure 2:**
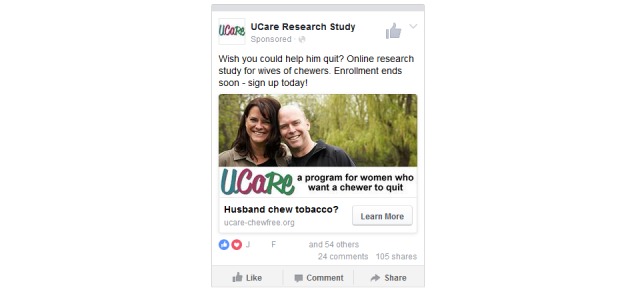
Sample Facebook ad.

For our ad title, we used “Husband chew tobacco?” throughout the study (which may now violate the policy just described), and we varied the main block of text to see what would work best. We also changed the text depending on the stage of the study. The first ads mentioned that the study was “new,” and toward the end of recruitment, we used “enrollment ends soon” as a method to motivate potential participants to take quick action. For ads in between, we found that “Everything he wishes you knew about quitting smokeless. Online research study – sign up now!” was especially effective. We tested ads that stated that participating in the study was “free,” but those ads had a low response rate. We carefully avoided mentioning the financial incentives we provide for completing follow-up surveys, either in the ad or on the study’s main marketing page (the home page), to avoid attracting fraudulent participants [36,37]; other studies have been able to mention financial incentives by using an insider knowledge check to screen for fraud (eg, when recruiting veterans, checking to see that rank and pay grade at discharge matched [19,23]). See Figure 2 for an example of our ad. Research teams may do well to conduct formative work to get feedback from target participants on sample ads, including both text and graphics, before the recruitment campaign.

As part of creating the ad, it is important to link the ad both to the study URL (the place where participants are directed when they click on the ad) and to the Facebook fan page.

The “create a similar ad” or “duplicate advert” function can be useful for a campaign that will be rotating among different audiences. Sometimes, the function allowed us to set up the same ad in a different ad set with minimal input, and at other times, we were required to enter all the details again. All the graphics uploaded for use in an ad campaign are saved in the campaign’s “image library” and can be accessed again easily.

Each ad will require Facebook approval, which typically takes less than an hour, although we did find that some would occasionally take up to 12 hours. The display often said the ad was approved immediately, but it was not actually approved until we received a notification later. Facebook has specific criteria for approving or denying ads to prevent illegal marketing of certain products or services. For researchers studying addiction, this can result in erroneous denial of ads. Once we had an ad denied for mentioning tobacco—the reviewer (whether human or automated) had assumed that we had fallen afoul of the ban on tobacco advertising. We were able to file an appeal, following instructions we received in the email from Facebook telling us about the denial, but processing the appeal took several days.

### Monitoring Facebook Users’ Interaction With the Ad

There are 2 aspects of responses to the ad that the researcher can attend to: Facebook activity related to the ad and participants enrolling in the study. The Facebook notification feature will allow the researchers to see easily throughout the day when people are interacting with the ad with *likes*, *shares*, and *comments*, which typically include people *tagging* their friends to see the ad. The staff members who have been assigned as managers for the Facebook fan page and who are creating ads for the study will receive these notifications, which will give them clues regarding the popularity of the study and the times of days the ad is being shown.

In the comments on the ads, many women *tagged* specific friends, which gave us an implied endorsement from the tagger when the tagged woman saw the ad. Our study was unique and may have met a need that many potential participants had been expressing to their friends (eg, “I wish he’d quit!”); advertising on Facebook allowed us to capitalize on this. Many women also tagged their husbands to let them know about their interest in the study, and the husbands often responded. Many men encouraged their wives to sign up for the study or at least assured them that they would try to quit smokeless tobacco.

### Tracking Enrollment

If the study admin page allows for the researcher to see at a glance how many participants have enrolled, then monitoring enrollment will take a trivial effort; however, for greatest accuracy in tracking, this monitoring should be done at the same time daily. As mentioned earlier, an automated email with key enrollment figures can be sent to the research staff to facilitate tracking. We manually tracked the enrollment from each ad, recording the ad targeting and text, the dates, the number enrolling between those dates, and the number enrolling per day. We were able to enroll about 5.6 participants per day during the first 9 weeks of the study (the first pass through all geographic areas targeted for recruitment); the rest of the study averaged 1.9 participants per day.

### Strategies for Sustaining a Long-Term Advertising Campaign

Our primary campaign strategy was to rotate the ad among distinct geographic regions, focusing on 1 region at a time so that we could easily see how well each ad was doing (by monitoring the locations of enrollees on our admin page). We would typically give an ad at least 2 days to have an impact, and once people had begun enrolling in response to an ad, we would wait for responses to taper off and then move the ad once it seemed fairly dormant for 2 days. Facebook notifications of user interaction with the ad let us judge whether interest had truly dwindled or whether people were too busy to complete the enrollment on a given day.

We recruited throughout the United States and Canada. As noted above, we began by rotating through broad regions with a high prevalence of smokeless tobacco use. This phase of our campaign lasted about 2 months. Next, we began switching up the graphic and text and rotated through our broad regions again for 4 months. Then, we targeted very specific areas where our ad had done well for about 3 months, making sure to have approximately 15,000 to 40,000 in Facebook’s projected *reach* for the ad. To target specific areas, we used a map to identify the named communities in the region, which we entered into Facebook as a list. Facebook allows communities to be designated with a user-specified surrounding radius, allowing suburbs and more rural communities to be included or excluded. For some states with high numbers of smokeless tobacco users but low overall smokeless tobacco prevalence, we excluded the urban areas from the recruitment zone by specifying the names of the cities we wanted to exclude. (We did so anticipating that urban people would use up the budget if not excluded; see the discussion of ineligible or uninterested people clicking the ad in the Saturation: The Problem of Diminishing Returns section below). Researchers can use their own expertise to identify groups they would expect to respond to the ad and tailor their campaigns accordingly.

The time of year is important to take into account. September has historically been a good recruitment time for us, but for this study, September coincided with both the study launch and the “ends soon” ad campaign; therefore, we cannot conclude that the time of year was itself a key factor. January and early spring, like September, are also traditional *new beginnings* times and could potentially be good as well for an intervention focusing on changing health behaviors. Our recruitment was very low from mid-November to early January, even though we tried a boost of advertising in many areas simultaneously during early January to try to capitalize on New Year’s resolutions. The appeal to resolutions may be relevant for changing some behaviors such as quitting tobacco but did not appear to engage supporters. Researchers may want to consider the times of year that their prospective participants would be most responsive to their message.

By the time we had finished advertising in the very specific regions described above, we had accumulated around 1500 *likes* on our Facebook fan page, which allowed us to try 2 additional Facebook targeting options. The first of these was an advertisement that targeted Facebook friends of people who had liked the page, not limited by geographic region. This ad received a fairly high response rate: 32 participants in 13 days. Next, we used the “Lookalike” function in which Facebook used an internal algorithm to identify 1,000,000 people who they considered similar to the people who had liked our page. We then targeted members of this Lookalike sample, starting with an ad to all Lookalike sample members, then again going region by region (still specifying women aged 25 to 65 years and above who were married, etc, as described above). This strategy revitalized the campaign. To conclude the campaign, we returned to the most successful ads that included our “ends soon” message, rotating between them until we had reached our recruitment goal.

The “friends of people who like the page” and “Lookalike” options will be most useful when recruiting for a large study with a target audience that is demographically homogeneous (eg, wives of smokeless tobacco users). This type of advertising will be less useful for smaller studies and studies enrolling participants with health conditions that are relatively infrequent and randomly distributed. For example, a breast cancer patient is unlikely to have many friends who are also breast cancer patients, and people who are demographically similar to her will not be appreciably more likely to have breast cancer than people who are demographically different.

### Saturation: The Problem of Diminishing Returns

Perhaps the most important thing to know about Facebook advertising is this: although enrollment from each ad will taper off within a few days of its launch, people will continue to click on the ad (and use up your budget) every day. If you are paying per click rather than per impression (view), you are paying for these clicks. Whether these extra *clickers* are people who for whatever reason click on anything or bots who scour the internet to follow any available link [38-40], little can be done about the waste, other than to note that the ad will quickly see diminishing returns (reach saturation), and thus, it should be shown to a fresh target audience every few days.

It may be that fewer than 1% of the people who click on an ad will enroll in a study, especially if the study is a full-scale RCT. In our RCT, we enrolled 1145 people from 371,472 clicks, a rate of 0.3%. When we first began advertising on Facebook, the ratio of enrolled participants to clicks was much higher but still not more than about 1%. Others have had similar experiences: Ramo et al [29] converted 5875 clicks into 79 participants in an RCT for young adult smokers (1.3%), and Adam et al [18] converted 1001 clicks into 45 participants for a pregnancy-related RCT (4.5%). In our case, enrollment required first completing an eligibility screening, followed by informed consent, registration with personal contact information, and finally completion of a baseline survey that took perhaps 20 min to complete. This series of hurdles to enrollment may have discouraged many potential participants, but it also suggests that those who do complete the process may be more motivated to be actively involved in the intervention, which we consider a good trade-off. Simpler studies (eg, those requiring completion of a single survey) can get higher rates for converting Facebook ad clicks to participants (with some offering financial incentives and others not doing so). For example, Ramo and Prochaska [16] converted 14,808 clicks into 1548 completed surveys on tobacco and marijuana use (10.5%); Tan [17] converted 280 clicks into 59 completed surveys on math students’ learning preferences (21.1%). Conversely, Kapp et al [13] received 280 clicks on a Facebook ad to recruit women to complete a health survey on mammography, but no surveys were completed. A human papillomavirus study requiring an online questionnaire and a self-collected penile swab converted 41,811 clicks into 535 study completers (1.3%) [41].

Table 1 displays the recruitment results for our study, showing the numbers of people “reached,” clicking the link, beginning the enrollment process, eligible, consenting, and randomized. The *reach* and click data may be found in the Facebook Ad Manager, and the totals for beginning the process, eligible, consenting, and randomized were shown on the online administrative participant management system we had designed for this purpose. As the table indicates, Facebook displayed the ad to about 6.6 million women meeting our criteria, and 5.63% (371,472/6,600,839) of them clicked on the ad to see our study home page. A very small fraction of those then began the eligibility screening, and 30.63% (1554/5074) of those who began the eligibility screening completed it and were eligible to participate in the study. Of the eligible people, 96.99% (1416/1460) completed the informed consent, and 80.86% (1145/1416) of consenters provided their personal contact information, completed the lengthy baseline survey, and were randomized. Overall, 22.57% (1145/5074) of those who began the eligibility screening were randomized; 78.42% (1145/1460) of those who were eligible were randomized.

### Examining Ad Effectiveness

We analyzed the registration patterns associated with each of the 91 ads we used during the study. Almost 30% of participants were enrolled from the first 7 regional ads in approximately 2 months. The most successful ad in terms of total enrolled participants was the first ad targeting the Midwest, which yielded 95 participants from 13 days of advertising. After this period, from mid-October to late December, 10 ads yielded only 113 participants. The New Year’s simultaneous advertising plan (counted as a single ad for analyses) yielded 43 participants but was very expensive. Regional advertising was then hit-and-miss for several months, with some ads doing acceptably but others poorly. The “friends of friends” ad yielded 32 participants, and the Lookalike ads were moderately successful, with many yielding more than 15 participants but some far fewer.

**Figure 3:**
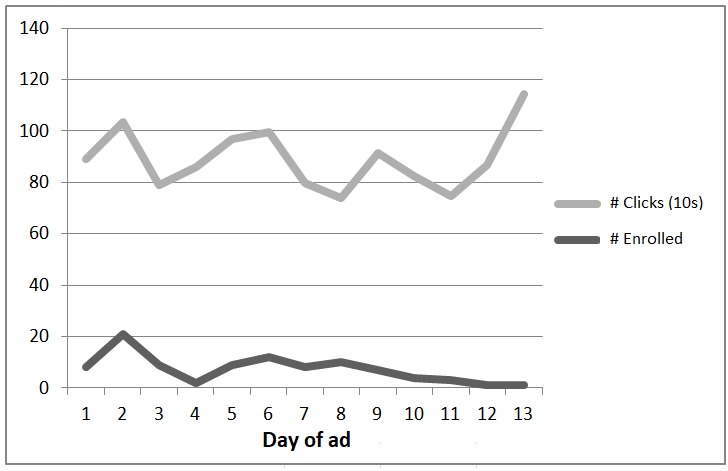
Enrollment versus ad clicks (tens of clicks) for 1 ad by day of ad.

Figure 3 displays the recruitment pattern from the Midwest ad yielding 95 participants. The figure illustrates 2 key points: enrollment gradually tapers off within a few days of launching even a successful ad like this one (the saturation effect), whereas ad clicks remain high throughout (700 to 1135 clicks per day). For this ad, enrollment dropped sharply on a holiday (day 4, Labor Day) but resumed thereafter; we discontinued the ad after day 13.

The vast majority of the participants (94.2%) lived in the target area for an ad and enrolled while the ad was running or within 2 days of its conclusion. Of the others, 2.2% could plausibly have been stragglers from an earlier ad (enrolling within several weeks of an ad beginning in their region and assigned an identification number based on that ad period) and were attributed to that ad. The other 3.6% lived outside the current ad’s target area (sometimes nearby but sometimes on the other side of the country), and we expect that most of these people were tagged or otherwise notified of the study by a friend seeing the ad; we attributed their enrollments to the current or most recent ad. Enrollment was consistent across the days of the week, from a low of 13.0% of the total sample enrolling on a Thursday to a high of 15.7% enrolling on a Sunday. Ads were placed on each day of the week, ranging from 6 ads that began on Sundays to 20 ads that began on Fridays.

**Table 1:** From “Reach” to randomization: UCare study recruitment data.

Enrollment step	Number	Proportion continuing from prior step
“People Reached”	6,600,839	Total viewing Facebook ad
Link (ad) clicks	371,472	5.63% of “reached”
Began online eligibility screening	5074	1.37% of clicks
Completed online eligibility screening	1554	30.63% of began screening
Eligible	1460	93.95% of completed screening
Consenting	1416	96.99% of eligible
Randomized	1145	80.86% of consenting

Smokeless tobacco prevalence data are available by state. We designed our study admin page to provide a downloadable Excel file with participant registration data, including their mailing addresses. These data allowed us to calculate per capita response rates by state to see which states were most welcoming (very simply, state adult population x smokeless tobacco user rate=estimated smokeless tobacco users in that state, divided by women enrolled from that state=state enrollment rate). For example, 33 women enrolling from Idaho gave us our best rate, at 47 per 100,000 smokeless tobacco users; 11 women enrolling from Texas gave us our worst rate, at 1 per 100,000 smokeless tobacco users. (As our previous smokeless tobacco cessation study [42] enrolling chewers directly had been very popular in Texas, we made extra efforts to improve recruitment of women there, but with no success until we were able to run a “lookalike” ad, as described above.) By tracking this type of information, researchers can target geographical regions that may be more responsive than others or take extra steps to enroll participants from audience subsections that are lagging behind expectation.

If a particular ad does poorly, it may reflect a lack of interest among the targeted group. This may be temporary (eg, severe local weather conditions). It may also reflect a decision by Facebook on how much to promote the ad, for example, if they deem this ad has “too much” text (although it may be identical to an ad recently used successfully). The same ad could be tried again in a few weeks, or a new (and if desired, essentially identical) ad could be created to see if the results improve.

### Advertising Costs

This advertising campaign resulted in a cost of US $112.48 per randomized participant (US $0.35 per click). In our pilot 2010 Facebook recruitment campaign with the same study population, we spent just under US $70 per participant. As the cost per click for that campaign is no longer available, we are unable to discern whether the rising cost was due to lower rates of converting clicks to participants or higher advertising prices. In their 2016 review of Facebook recruitment for health and psychosocial research, Thornton et al [34] reported that 110 studies had experienced costs per participant ranging from US $1.36 to US $110 (mean=US $17.48, SD US $23.06). Whitaker et al’s review of 35 studies found a median value of US $14.41 per health study participant recruited using Facebook [6]. The magnitude of the difference between these costs and our own may be attributable in part to the fact that most of these studies were not RCTs, and the burden of enrollment in terms of time, effort, and commitment would thus be far smaller. These studies could also be targeting populations that are easier to reach than those targeted in our study. Our cost of US $112 per participant is in line with the costs we incurred for using traditional advertising media in 2004 to 2005 (US $92 per participant for free publicity in newspaper, radio, and TV news and US $115 per participant for newspaper display ads); in that study, Google AdWords was far more cost-effective (US $7 per participant). None of these advertising methods would have allowed us to exclude men from seeing the study and attempting to enroll, an outcome we wanted to avoid.

The costs for recruiting our population may not be representative of other groups. Pedersen et al recruited 793 young veteran drinkers to a *very brief* intervention in only 7 days for less than US $5 per participant [23]. For this study, 4.4% of clicks were converted to participants, in comparison with our rate of 0.3%.

### Ethical Considerations

There are 2 important ethical concerns to using Facebook advertising: Facebook’s potential use of the data and the risks from interacting with social networks. When a user clicks on an ad, and especially when they *like* an ad or a campaign’s Facebook page, information about these choices is stored by the user’s browser cookies [19], and Facebook’s algorithms may conclude that the user has an interest in the topic and begin showing the user related links. This may make the user uncomfortable, and they may conclude that the study is violating their privacy. One way to partially mitigate this risk would be to include text on the study’s fan page cautioning the user that *liking* the page or clicking on the embedded link to the study’s own homepage may be used by Facebook marketing. Visitors could be provided with a nonhypertext URL to the study, with the suggestion that they access the study by copying and pasting the URL to a new browser tab. This option may be weighed against the risk to the study of reducing its page *likes*, which is an important element of a long-term advertising campaign, as described above.

The risks from interacting with social networks are largely related to privacy, especially for stigmatizing health conditions. When someone sees an ad and *tags* a friend who they think may be interested, others can see this tagging and will assume there is some association between the tagged person and the condition. Unless Facebook provides a way to eliminate tagging (eg, by disabling comments on the ad), there is no realistic way to avoid this risk other than not advertising for this population. One study recruiting for a domestic violence intervention took 2 measures to protect potential participants’ safety: placing their ads at the side of the screen rather than in the user’s newsfeed and asking women’s organizations to post the ad for them such that women would already be following those pages to receive the information about the study in their newsfeed. They also included accompanying text for all ads, “Please open the link in a new browser window” and “Share only if safe to do so” [43].

Detailed ethical guidelines for protecting users’ privacy during social media recruitment have been developed by Bender et al [44], using Privacy by Design principles.

### Key Points for Using Facebook Advertising

Following are our key recommendations for using Facebook advertising:

- First, decide whether Facebook is really an optimal advertising medium for the study.
- For a large-scale RCT, a substantial advertising budget may be necessary.
- Before launching recruitment, design an infrastructure for enrolling and managing participants, including a simple method to monitor the success of each ad daily.
- Before beginning a Facebook advertising campaign, create a fan page on Facebook for the study to connect with the ad; plan to keep the page active with regular posts.
- Be aware that Facebook changes its ad requirements often. Consider getting feedback from targeted participants on the text and graphics of ads before using them.
- Plan for each Facebook ad to reach saturation quickly. Design the ad campaign with a specific rotation strategy in mind (eg, geographic region or a demographic variable), and create an easy way to track enrollment by this variable to help monitor each ad’s effectiveness.
- The “friends of people who like the page” and “Lookalike” options will be most useful when recruiting for a large study with a target audience that is demographically homogeneous but less so for a target audience whose members are heterogeneous, for example, those diagnosed with a particular low-incidence disease.
- Monitor the advertising campaign regularly to avoid overcommitting the advertising budget and be flexible and ready to change strategies if the responses are not as expected.
- Above all, be aware that a great many people will click on Facebook ads without the intention of enrolling in the study, and an ample budget will be necessary.

### Conclusions

Our experiences have shown that it is possible to recruit a moderately large RCT sample via Facebook. This recruitment method provides considerable flexibility to monitor and modify the advertising tactics based on feedback. In our case, we were advertising for participating in a study that would be very appealing to potential participants if they became aware of it and that was easy for people to promote to their Facebook friends who they knew may also be interested. This method of recruitment may not work for all types of projects or participant populations. Furthermore, Facebook and other social media are in a constant state of flux, and the methods described in this paper may not work the same way in the future.

## Abbreviations

eHealth: electronic health
RCT: randomized clinical trial
